# Systematic Review of the Impact of Transcranial Direct Current Stimulation on the Neuromechanical Management of Foot and Ankle Physical Performance in Healthy Adults

**DOI:** 10.3389/fbioe.2020.587680

**Published:** 2020-10-30

**Authors:** Songlin Xiao, Baofeng Wang, Xini Zhang, Junhong Zhou, Weijie Fu

**Affiliations:** ^1^School of Kinesiology, Shanghai University of Sport, Shanghai, China; ^2^The Hinda and Arthur Marcus Institute for Aging Research, Hebrew SeniorLife, Boston, MA, United States; ^3^Harvard Medical School, Boston, MA, United States; ^4^Key Laboratory of Exercise and Health Sciences of Ministry of Education, Shanghai University of Sport, Shanghai, China

**Keywords:** transcranial direct current stimulation, foot, ankle, physical performance, neural circuitry, neuromechanical management

## Abstract

**Objective:** This study aims to review existing literature regarding the effects of transcranial direct current stimulation (tDCS) on the physical performances of the foot and ankle of healthy adults and discuss the underlying neurophysiological mechanism through which cortical activities influence the neuromechanical management of the physical performances of the foot and ankle.

**Methods:** This systematic review has followed the recommendations of the Preferred Reporting Items for Systematic reviews and Meta-Analyses. A systematic search was performed on PubMed, EBSCO, and Web of Science. Studies were included according to the Participants, Intervention, Comparison, Outcomes, and Setting inclusion strategy. The risk of bias was assessed through the Cochrane Collaboration tool, and the quality of each study was evaluated through the Physiotherapy Evidence Database (PEDro) scale.

**Results:** The electronic search resulted in 145 studies. Only eight studies were included after screening. The studies performed well in terms of allocation, blinding effectiveness, and selective reporting. Besides, the PEDro scores of all the studies were over six, which indicated that the included studies have high quality. Seven studies reported that tDCS induced remarkable improvements in the physical performances of the foot and ankle, including foot sole vibratory and tactile threshold, toe pinch force, ankle choice reaction time, accuracy index of ankle tracking, and ankle range of motion, compared with sham.

**Conclusion:** The results in these studies demonstrate that tDCS is promising to help improve the physical performances of the foot and ankle. The possible underlying mechanisms are that tDCS can ultimately influence the neural circuitry responsible for the neuromechanical regulation of the foot and ankle and then improve their physical performances. However, the number of studies included was limited and their sample sizes were small; therefore, more researches are highly needed to confirm the findings of the current studies and explore the underlying neuromechanical effects of tDCS.

## Introduction

The functionality and physical performances of foot and ankle, including muscular strength, somatosensory function, and endurance, play a key role in locomotor control when standing, walking, jumping, and endurance running in everyday activities (Rodgers, 1988; Aagaard, 2018). In addition to the peripheral nervous system, the cortical functional networks of the brain have been linked to the formation and regulation of the physical performances of the foot and ankle (Noakes, 2011; Foerster et al., 2018a). A decrease in the excitability of cortical regions is associated with diminished biomechanical management of physical performance and increases the risk of injuries (e.g., chronic ankle inability) (Needle et al., 2017). Therefore, strategies designed to facilitate the neural circuitry of the brain have a great potential of improving the functional and physical performances of the foot and ankle.

One promising approach is transcranial direct current stimulation (tDCS). TDCS non-invasively modulates the excitability of brain regions by delivering low-amplitude current flow between two or more electrodes placed on the scalp (Nitsche and Paulus, 2000, 2001; Reardon, 2016). Anodal tDCS can increase cortical excitation through the tonic depolarization of the membrane resting potential, and cathodal tDCS may decrease cortical excitation by the hyperpolarization of the membrane resting potential (Stagg and Nitsche, 2011; Rahman et al., 2013). TDCS can enhance the cognitive–motor function and is beneficial for multiple neurological and psychiatric disorders (e.g., depression and Alzheimer's disease) (Kuo et al., 2014; Summers et al., 2016).

In recent years, researchers have explored the effects of tDCS on the physical performance of healthy individuals. Anodal tDCS applied over the primary motor cortex (M1) can improve multiple physical performances, such as balance control (Saruco, 2017; de Moura et al., 2019), pain perception (Vaseghi et al., 2014), muscle strength, and muscular endurance (Lattari et al., 2018; Vargas et al., 2018; Machado, S. et al., 2019). Specifically, tDCS designed to target the sensorimotor region has induced improvements in the physical performances of the foot and ankle of healthy adults (Devanathan and Madhavan, 2016; Zhou et al., 2018). For example, Zhou et al. (2018) observed that one session of tDCS targeting M1 has improved the vibrotactile sensation of the foot sole of healthy older adults when standing. These efforts have shed a light on a novel strategy for the enhancement of the physical performances of the foot and ankle by using tDCS to modulate the excitability of cortical brain regions. Considering the different methodologies and unpredicted effects, systematic review of published studies can provide valuable summaries of the effects of tDCS on the physical performances of the foot and ankle.

This study thus aims to systematically review available peer-reviewed publications to date on the effects of tDCS on the physical performances of the foot and ankle of healthy adults. We then further discuss the underlying neurophysiological mechanism through which cortical activities influence the neuromechanical management of physical performances of the foot and ankle. This review will provide the most recent achievements and a better understanding of research efforts in this direction to ultimately help optimize the implementation of tDCS to enhance the physical performances of the foot and ankle in the near future.

## Methods

The method of this review was designed following the recommendations of the Preferred Reporting Items for Systematic reviews and Meta-Analyses and the Cochrane Handbook for Systematic Reviews of Interventions (Moher et al., 2009; Cumpston et al., 2019).

### Search Strategy

This systematic review conducted a comprehensive search of three databases, namely, PubMed, Web of Science, and EBSCO, up to May 2020. The search was performed using the terms “foot,” “toe,” and “ankle,” which were separately combined with “transcranial direct current stimulation” or “tDCS” in all databases. Boolean operators “AND” and “OR” were used to combine keywords according to the recommendations of each database. All results found in the search were imported into the EndNote reference manager (EndNote X9, USA, Stanford) to gather together and automatically find out duplicate records.

### Eligibility Criteria and Article Selection

**Figure 1:**
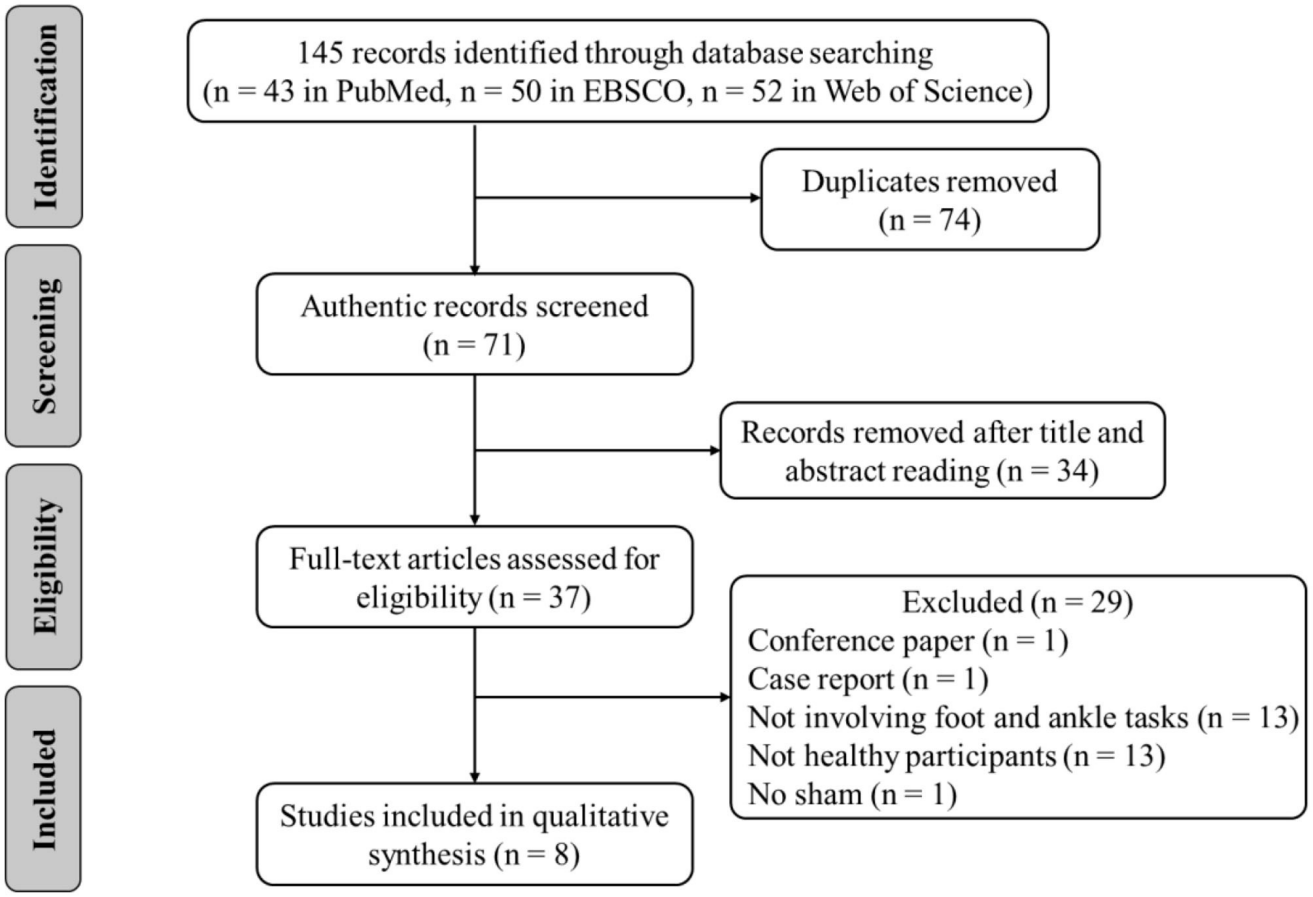
Flow diagram of the search strategy.

The following inclusion criteria were applied based on the Participants, Intervention, Comparison, Outcomes, and Study design (Ferreira et al., 2019). (1) The participants were healthy adults with no history of musculoskeletal injury and overt neurological disease. (2) The intervention was tDCS, regardless of stimulation types, stimulus intensity, duration, and electrode location. (3) A comparison was with sham tDCS (i.e., placebo). (4) The primary outcomes were strength, perception, flexibility, or other related variables of the foot and ankle. (5) The study design was randomized, crossover, and sham-control designs. Animal studies and non-English studies were excluded. Reviews, case reports, letters, opinions, and conference abstracts were also excluded (Figure 1).

Two researchers independently evaluated the results of the search and resolved differences through discussion (SL and BF). The Abstracts and full texts of relevant articles were read thoroughly, and only those that met the eligibility criteria were selected. Then, the researchers further confirmed the selected articles and discussed possible disagreements; if any disagreement persisted, then a third researcher was consulted and judged the results (WJ).

### Data Extraction

The original data of the included articles were summarized in Microsoft Excel (Microsoft Corporation, Redmond, WA, USA). The findings were divided into two categories, namely, the effects of tDCS on the physical performances of (1) the foot and (2) ankle, to facilitate the interpretation of the results. Besides, the following data were summarized: author, sample size, gender, age, anodal/cathodal location, electrode size, current intensity, duration, control, and main outcomes.

### Quality and Risk-of-Bias Assessments

The Physiotherapy Evidence Database (PEDro) scale was used to assess the quality of each study (Maher et al., 2003). Studies with a PEDro score of <6 were deemed as having low quality.

The risk of bias of each study was assessed using the Collaboration's “Risk of Bias” tool, version 5.2 based on the Cochrane Handbook for Systematic Reviews of Interventions (Cumpston et al., 2019). The risk of bias for each study was judged as “low,” “high,” or “unclear” risk of bias. The two researchers independently evaluated the PEDro score and risk of bias of each study; a third researcher was consulted to reach a final consensus if any disagreement persisted.

## Results

A total of 145 related articles were found in the databases (43 in PubMed, 50 in EBSCO, and 52 in Web of Science). Only eight articles were included for systematic review after removing duplicate articles and excluding irrelevant studies by reading the titles, Abstracts, and full texts. Four studies examined the effects of tDCS on foot physical performance (Table 1), and the other four studied its effects on ankle physical performance (**Table 3**).

**Table 1:** The characteristics of studies investigating the effect of tDCS on foot physical performance.

**Study**	**Sample**	**Gender**	**Age (years)**	**Drop-outs (N)**	**Design**	**Task**	**Cortical excitability examined**	**PEDro score**
Yamamoto et al. (2020)	10	10 males	22–34	None	Crossover	Foot somatosensory tests	No	9
Xiao et al. (2020)	14	14 males	22.8 ± 1.2	None	Crossover	Foot flexor strength task	No	9
Zhou et al. (2018)	20	20 males	61 ± 4	None	Crossover	Foot sole vibratory sensation task	No	9
Tanaka et al. (2009)	10	8 males 2 females	20–35	3	Crossover	Toe pinch force task	No	6

### Effects of tDCS on Foot Physical Performance

Four studies investigated the effect of tDCS on foot physical performance. Fifty-four participants, consisting of 52 males and 2 females, were recruited with an age of between 20 and 61 years (Table 1). A bias in gender was observed across these studies.

Only the immediate effects of one-session tDCS were examined in these studies. Three studies used conventional tDCS applied over the sensorimotor cortex with an electrode size of 35 cm^2^ (Tanaka et al., 2009; Zhou et al., 2018; Yamamoto et al., 2020). One study used a 4 × 1 ring high-definition tDCS (HD-tDCS), in which the anodal electrode was placed over Cz and was surrounded by four cathodal electrodes with a size of 1 cm^2^ (Xiao et al., 2020). Three studies applied the current intensity of 2 mA (Tanaka et al., 2009; Zhou et al., 2018; Xiao et al., 2020), and one study applied the intensity of 1.5 mA (Yamamoto et al., 2020). The duration of tDCS was 10 min in two studies (Tanaka et al., 2009; Yamamoto et al., 2020) and 20 min in the other two studies (Zhou et al., 2018; Xiao et al., 2020). The current density at the stimulation electrode was 0.043 or 0.057 mA/cm^2^ with a total charge between 0.026 and 0.069 C/cm^2^. Sham was used as a control in these studies, in which the placements of electrodes were the same as real tDCS but the current was delivered in only the first 30 or 60 s of the stimulation (Table 2).

**Table 2:** Stimulation protocols and main outcomes of studies investigating the effect of tDCS on foot physical performance.

**Study**	**Anodal/cathodal location**	**Electrode size (cm^**2**^)**	**Current (mA)**	**Duration (min)**	**Total charge (C/cm^**2**^)**	**Control**	**Main outcomes**	**Significant effect vs. sham**
Yamamoto et al. (2020)	C: Left C3 R: Right supraorbital region	35	1.5	10	0.026	Sham: 30 s	↓ Tactile threshold of distal pulp of the hallux	c-tDCS > sham
Xiao et al. (2020)	A: Cz R: C3, C4, Fz, Pz	1	2.0	20	NR	Sham: 30 s	→ Foot flexor strength; ↓ ankle INV/DF kinesthesia threshold	Post- > pre- in the two conditions;≠ between the conditions
Zhou et al. (2018)	A: Left C3 R: Right supraorbital region	35	2.0	20	0.069	Sham: 60 s	↓ Standing vibratory threshold of foot sole	a-tDCS > sham
Tanaka et al. (2009)	A and C: M1 (“hotspot” of the TA muscle) R: Right forehead	35	2.0	10	0.034	Sham: 30 s	↑ Toe pinch force	a-tDCS > sham

*A, anodal; C, cathodal; R, reference electrode; M1, primary motor cortex; TA, tibialis anterior; a-tDCS, anodal transcranial direct current stimulation; c-tDCS, cathodal transcranial direct current stimulation; INV, inversion; DF, dorsiflexion; PEDro, Physiotherapy Evidence Database (PEDro) scale; ↓, denotes a decrease; → , denotes no significant change; ↑, denotes an increase; C3, C4, Cz, Fz, Pz: the electrodes placement of the 10/20 EEG system; NR, not reported*.

Several variables were assessed in these studies, including foot sole standing vibratory threshold, foot tactile threshold, foot flexor strength, and toe pinch force. Specifically, Zhou et al. (2018) observed that the standing vibratory threshold was decreased (i.e., better sensation) after anodal tDCS compared with sham. Yamamoto et al. (2020) examined the effects of cathodal tDCS designed to target the left motor area on foot tactile threshold and observed a significant decrease in the tactile threshold of the left center of the distal pulp of the hallux after cathodal tDCS compared with sham. One study showed that tDCS could improve toe pinch force (Tanaka et al., 2009). More recently, Xiao et al. (2020) found no significant differences in foot flexor strength between anodal HD-tDCS and sham stimulation.

### Effects of tDCS on Ankle Physical Performance

Four studies examined the effects of tDCS on ankle physical performance. Forty-four participants, consisting of 25 males and 19 females, were recruited. The age of the participants was between 18 and 32 years (Table 3).

**Table 3:** The characteristics of studies investigating the effect of tDCS on ankle physical performance.

**Study**	**Sample**	**Gender**	**Age (years)**	**Drop-outs (N)**	**Design**	**Task**	**Cortical excitability examined**	**PEDro score**
Mizuno and Aramaki, (2017)	10	10 males	25 ± 3	2	Crossover	Passive dorsiflexion task	No	9
Devanathan and Madhavan, (2016)	14	6 males 8 females	20–32	None	Crossover	Ankle reaction time task	No	9
Sriraman et al. (2014)	12	4 males, 8 females	20–32	None	Crossover	Ankle motor task	Yes	6
Shah et al. (2013)	8	5 males, 3 females	18–26	None	Crossover	Ankle visuomotor task	Yes	6

Only the immediate effects of one-session tDCS were examined in these studies. The target of tDCS was M1 in three studies (Sriraman et al., 2014; Devanathan and Madhavan, 2016; Mizuno and Aramaki, 2017), and the targets in the other study were M1 and the cerebellum (Shah et al., 2013). Three studies used transcranial magnetic stimulation to identify the area of M1 by hot spotting for the left tibialis anterior (TA) muscle (Shah et al., 2013; Sriraman et al., 2014; Devanathan and Madhavan, 2016), and one study placed the electrodes according to the 10/20 EEG system (Mizuno and Aramaki, 2017). The electrode sizes were between 8 and 35 cm^2^ (Shah et al., 2013; Sriraman et al., 2014; Devanathan and Madhavan, 2016; Mizuno and Aramaki, 2017). Three studies applied the intensity of 1 mA for 15 min (Shah et al., 2013; Sriraman et al., 2014; Devanathan and Madhavan, 2016) and the other one used 2 mA for 10 min (Mizuno and Aramaki, 2017). The current density at the stimulation electrode was 0.057, 0.08, or 0.125 mA/cm^2^ with a total charge between 0.034 and 0.113 C/cm^2^. Sham was used as a control in these studies, in which the placement of electrodes was the same as real tDCS but the current was delivered in only the first 30 s of the stimulation.

For the excitability assessment induced by tDCS, one study showed that cerebellar cathodal and anodal tDCS increased the normalized motor-evoked potential (MEP) amplitudes for the TA muscle compared to the sham condition (Shah et al., 2013). The other study observed a trend toward a greater change in MEP amplitude during anodal tDCS compared to the pre-stimulation (Sriraman et al., 2014) (Table 4).

**Table 4:** Stimulation protocols and main outcomes of studies investigating the effect of tDCS on ankle physical performance.

**Study**	**Anodal/cathodal location**	**Electrode size (cm^**2**^)**	**Current (mA)**	**Duration (min)**	**Total charge (C/cm^**2**^)**	**Control**	**Main outcomes**	**Significant effect vs. sham**
Mizuno and Aramaki, (2017)	A and C: Cz R: Center of the forehead	35	2.0	10	0.034	Sham: 30 s	**↑** Ankle range of motion	c-tDCS > sham
Devanathan and Madhavan, (2016)	A: M1 (“hotspot” of the TA muscle) R: Right supraorbital region	12.5	1.0	15	0.072	Sham: 30 s	↓ Ankle dorsiflexion choice reaction time	a-tDCS > sham
Sriraman et al. (2014)	A: M1 (“hotspot” of the TA muscle) R: Right supraorbital region	8	1.0	15	0.113	Sham: 30 s	**↑** Accuracy index of ankle tracking	a-tDCS > sham
Shah et al. (2013)	A and C: M1 (‘hotspot' of the TA muscle), left cerebellum R: Right supraorbital region, left buccinator muscle	8	1.0	15	0.113	Sham: 30 s	**↑** Accuracy index of ankle tracking	a-tDCS > sham cerebellar a-tDCS > sham cerebellar c-tDCS > sham

*A, anodal; C, cathodal; R, reference electrode; M1, primary motor cortex; TA, tibialis anterior; a-tDCS, anodal transcranial direct current stimulation; c-tDCS, cathodal transcranial direct current stimulation; PEDro, Physiotherapy Evidence Database (PEDro) scale; ↓, denotes a decrease; ↑, denotes an increase; Cz, the electrodes placement of the 10/20 EEG system*.

Ankle physical performance outcomes, including ankle reaction time, ankle range of motion, accuracy index for the ankle motor task, and passive kinesthesia thresholds, were measured in these studies. Devanathan and Madhavan (2016) observed that anodal tDCS could reduce the choice reaction time of ankle dorsiflexion compared with a sham condition. Mizuno and Aramaki (2017) observed that cathodal tDCS significantly increased ankle range of motion. Shah et al. (2013) and Sriraman et al. (2014) explored the effects of tDCS on ankle motor skill learning. The former found that anodal cerebellar, cathodal cerebellar, and anodal M1 tDCSs improved the target-tracking accuracy index of the ankle; and the latter similarly observed that tDCS increased motor performance during the practice of a skilled ankle motor task. Xiao et al. (2020) observed that anodal HD-tDCS decreased the passive kinesthesia thresholds of ankle inversion and dorsiflexion from pre- to post-stimulation, but no significant differences were observed between anodal and sham HD-tDCS.

### Risk of Bias and Quality of Evidence

**Figure 2:**
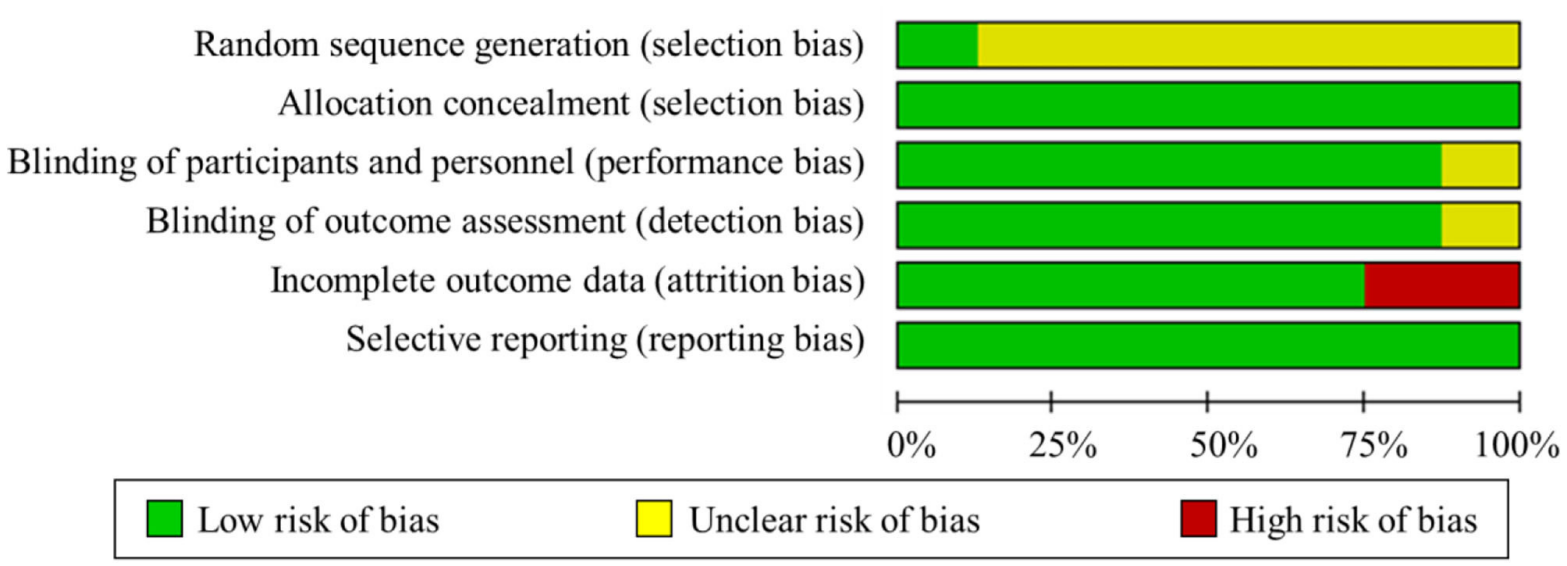
Summary of risk-of-bias assessment.

The summary risk of bias graph was illustrated in Figure 2. One study maintained a low risk of bias in all the domains evaluated (Xiao et al., 2020), whereas the other studies showed a certain high or uncertain risk in the risk-of-bias assessment. Regarding random sequence generation, all studies utilized randomization, but only one study sufficiently described the methods used for random sequence generation (Xiao et al., 2020). For blinding, five studies used a double-blind approach, one utilized single blinding, and the remaining described that no one noticed the difference between groups. Only two studies presented a high risk of incomplete outcome data. Three participants did not perform the hand pinch force task because of machine trouble and some other reasons in Tanaka et al. (2009). Two participants were excluded based on the results of the Smirnov–Grubbs rejection test by (Mizuno and Aramaki, 2017). Regarding selective reporting, all studies were deemed as having a low risk of bias. Besides, the PEDro scores of all the studies were over six, which indicated that all the included studies have high quality. Generally, the studies performed well in terms of allocation, blinding effectiveness, selective reporting, avoidance of order effects, maintenance of stimulation well-tolerated, and absence of side effects.

## Discussion

In this study, we systematically reviewed the literature reporting the effects of tDCS on the physical performances of the foot and ankle. Seven of the eight included studies showed improvements in the task performances of the foot and ankle, such as motor performance, motor learning skill, and somatosensory function, which suggests that tDCS is promising to help improve the physical performances of the foot and ankle. However, due to the small sample size, various tDCS parameters, etc., these findings still need to be examined and confirmed in future studies. We will thus discuss the underlying mechanism and outlook on the possible directions of futures studies.

Nowadays, the effectiveness of tDCS is gradually certified in improving physical performances (Vargas et al., 2018; Lattari et al., 2020a,b). The effects of tDCS on lower limb performance, especially the physical performances of the foot and ankle, are relatively understudied to date (Fleming et al., 2017). Applying tDCS over the foot and ankle area of the motor cortex is challenging because of its deep position in the interhemispheric fissure (Foerster et al., 2018b). However, an initial study exploring the effects of tDCS on lower limb M1 excitability reported that 10 min of 2 mA anodal conventional tDCS with an electrode size of 35 cm^2^ can increase the corticospinal excitability of the leg area of the motor cortex as reflected by an increase in the amplitude of motor-evoked potentials of the TA muscle (Jeffery et al., 2007). In line with the aforementioned preliminary finding, subsequent studies have shown that tDCS can also modulate the excitability of the lower-limb area of the motor cortex and further alter the physical performances of the foot and ankle of healthy adults and thus has potential benefits on promoting the physical performances of the foot and ankle (Tanaka et al., 2009; Shah et al., 2013; Sriraman et al., 2014).

Notably, high variability in the effects of tDCS on these types of physical performances was observed (Angius et al., 2018). For example, anodal tDCS failed to enhance the muscle strength of the lower extremity of healthy participants, including knee extensor and foot flexor strength (Flood et al., 2017; Maeda et al., 2017; Xiao et al., 2020). The variability may have arisen from the variance in the characteristics of the participants, the design of study protocols, and tDCS montages. Specifically, the variance in experimental outcomes in response to tDCS was caused by several factors associated with high inter-individual variability, including the organization of local circuits, basal level of function, psychological state, level of neurotransmitters and receptor sensibility, baseline neurophysiological state, and genetic aspects (López-Alonso et al., 2015; Pellegrini et al., 2018a,b; Machado et al., 2019). In addition, those previous studies used different experimental designs and stimulation protocols (i.e., different montages), potentially contributing to the variance in the results or even the reversal effect (Datta et al., 2009; Angius et al., 2018; Machado, S. et al., 2019; Hassanzahraee et al., 2020; Pellegrini et al., 2020). For example, Hassanzahraee et al. (2020) observed the reversal of corticospinal excitability of anodal tDCS with a current intensity of 1.0 mA when the stimulation duration was over 26 min, which may provide certain implications for preventing excessive brain activation. Additionally, a “ceiling effect” may limit the benefit induced by tDCS in healthy adults (Zhou et al., 2018), that is, healthy participants can perform the task well at baseline; thus, the tDCS-induced improvement would be not as high as expected. Nevertheless, the main outcomes of these studies showed the potential of tDCS in enhancing the physical performances of the foot and ankle, yet its effectiveness should be further confirmed in future studies with more rigorous study design and larger sample sizes.

These studies showed the potential of tDCS in enhancing the physical performances of the foot and ankle. However, the montages of tDCS (e.g., current intensity, duration, and electrode size) were inconsistent across the included studies. Specifically, three studies that investigated the effect of tDCS on foot physical performance used the conventional tDCS, which delivered a current of 1.5–2 mA via two large sponges (size: 35 cm^2^). The total charge of received current on targets was between 0.026 and 0.069 C/cm^2^, which can adequately induce variation in the response threshold of the stimulated neurons and change the electrical activity of the neurons (Fertonani and Miniussi, 2017). Thus, Zhou et al. (2018) found that 20 min of 2 mA tDCS applying over C3 can also improve foot somatosensory function. Similarly, among the studies investigating the effect of tDCS on ankle physical performance, four studies used the conventional tDCS delivering the current intensity between 1 and 2 mA via two large electrodes with different sizes (i.e., 8–35 cm^2^). The total current charge was between 0.034 and 0.113 C/cm^2^, and these studies showed that applying tDCS over M1 could increase ankle motor performance. However, this conventional tDCS may induce diffuse current flow on targets and the distribution of the induced electrical field may vary across participants. More importantly, the return of current in this conventional tDCS cannot be well-controlled, and may thus induce the inhibitory effects on the cortical regions next to the targeted area (To et al., 2016). For example, Zhou et al. (2018) targeted the primary sensory cortex (C3 area of 10/20 EEG template) using large sponge electrodes, and the excitability of the primary motor cortex (located anatomically next to the primary sensory cortex) may be inhibited, which may thus limit the effects of tDCS on the functional performance (e.g., they did not observe the significant improvement in the performance of timed-up-and-go test of mobility, though the foot-sole tactile sensation was improved). Besides, only one study using HD-tDCS observed a significant improvement in foot physical performance from pre- to post-stimulation, but not between anodal and sham HD-tDCS (Xiao et al., 2020). For anodal HD-tDCS, the anodal electrode was set at 2 mA, while the four return electrodes placed 3.5 cm apart from the anodal electrode were programmed to −0.5 mA. The previous study anticipated that −0.25 mA inhibitory input from the surrounding cortical areas may negate or override the focalized 1 mA current intensity over M1, which may influence overall M1 excitability (Pellegrini et al., 2020). Thus, the improvements in foot physical performance induced by anodal HD-tDCS may not be significant (Xiao et al., 2020). The applicable HD-tDCS protocol and its effectiveness should be further confirmed in future studies with more rigorous study design and larger sample size.

The mechanisms of tDCS in improving the physical performances of the foot and ankle are still uncertain (Angius et al., 2017). However, several possible explanations have been made. The modulation of the cortical excitability and the activation of the synaptic neuroplasticity of targeting brain regions play a key role in the regulation of neural circuits responsible for biomechanical management (Nitsche et al., 2008). Specifically, the current flow delivered by tDCS may augment active synaptic connections between the neuronal structures of cortical regions and thus may induce sustained changes in the neural activity of motor neurons. Such neuronal changes can improve the degree of the synchronous discharge of motor units, which influence the neural circuitry on the neuromechanical regulation of the foot and ankle and then improve their physical performance (Bindman et al., 1964; Dutta et al., 2015; Patel et al., 2019). TDCS can also induce an increase in cortico-muscular coherence, which is defined as the coherence mapping the synchrony between cortical neural activity and muscles (Chen C. et al., 2019). This occurrence may also support the aforementioned hypothesis of the underlying mechanism. Besides, the neuronal circuits involved in the regulation of motor tasks are likely active at a heightened state (Ziemann et al., 2004; Sriraman et al., 2014; Chen J. et al., 2019). The tDCS-induced improvements in physical performance and/or motor learning skills are commonly observed in the presence of activity-dependent modifications of synapses. Thus, the membrane shifting induced by anodal tDCS accessibly may shape synaptic plasticity and improve motor performance and learning (Sriraman et al., 2014). Sensory sensitivity depends on the degree of excitation in the somatosensory cortex and the integrity of the individual's neural circuitry (Fregni and Pascual-Leone, 2007). Wang et al. (2015) observed that anodal tDCS could modulate and augment cortical responsiveness to foot pressure stimuli in healthy adults by increasing the activation of the left posterior paracentral lobule (including the primary sensory cortex [S1]), and this tDCS-induced increase in cortical responsiveness to pressure stimuli may help the sensation of the lower limbs. Additionally, S1 is next to M1; therefore, tDCS targeting S1 may also increase the excitability of M1. Thus, improvements in sensory function may be attributed to the tDCS-induced activation of M1.

Interestingly, cathodal tDCS applied over the motor area also induced a positive effect on foot tactile sensation and ankle range of motion (Mizuno and Aramaki, 2017; Yamamoto et al., 2020). Cathodal tDCS usually decreases cortical excitability. By contrast, the administration of cathodal tDCS to the motor cortex of one hemisphere increases the excitability of the motor cortex of the other hemisphere in healthy participants (Schambra et al., 2003) and improves the physical performance of the ipsilateral foot (Yamamoto et al., 2020). Besides, an increase in ankle range of motion may be based on decreased pain perception secondary to the decreased excitability of the cerebral cortex caused by cathodal tDCS (Mizuno and Aramaki, 2017). Although anodal and cathodal tDCS maintain discrepant neurophysiological effects, both have shown benefits in improving the physical performance of the foot and ankle.

Multiple intermediate structural and functional levels between cortical regions and the foot and ankle in the regulatory pathway of the physical functions of the foot and ankle may mediate and integrate the effects of tDCS (Fertonani and Miniussi, 2017). These components are interconnected over multiple levels; therefore, tDCS that modulates neurons close to the discharge threshold, which are potentially engaged in the execution of a specific task, may also regulate the functionality of multiple components (e.g., task-related networks and secondary-level neural circuitry) in the regulatory system (Siebner et al., 2009; Bikson et al., 2013; Miniussi et al., 2013; Luft et al., 2014). However, some improvements in performance have been noted without remarkable changes in corticospinal excitability (Abdelmoula et al., 2016; Angius et al., 2016). Thus, characterizing the multi-level components of the regulatory system of the foot and ankle's physical function in future studies using neuroimaging techniques will ultimately advance the understanding of the underlying mechanisms behind the ergogenic neuromechanical effects of tDCS on the foot and ankle, as well as the other parts of the body extremities.

The results of this systematic review should also be taken with caution since the included studies have some methodological limitations. First, the sample size of the included studies in this review is relatively small. Second, the selection of tDCS parameters varied across studies, and many of them lacked optimization. For example, two studies applied the conventional tDCS over the C3 area, which may not be the appropriate target for the control of the lower-extremity function (Zhou et al., 2018; Yamamoto et al., 2020). Thus, a standardized protocol of tDCS using a more rigorous study design and advanced neuro-modeling technique is highly demanded in future studies aiming at examining the effectiveness of tDCS on the physical performances of the foot and ankle. Besides, the neurophysiological mechanisms through which tDCS benefits the physical performance are still not fully understood. Only two studies measured the changes of cortical excitability within the tDCS targets (i.e., M1), but the “dose-response” effects of tDCS were not examined (Shah et al., 2013; Sriraman et al., 2014). Further studies are warranted to estimate the on-target “does” of tDCS (e.g., the normal component of the electric field within the tDCS targets) using neuro-modeling techniques with the brain magnetic resonance images of participants and correlate such dose to the observed functional improvements (Fischer et al., 2017). This will provide fundamental knowledge into the causal relationship between the tDCS-induced neurophysiological changes of cortical regions and the changes in behavior induced by tDCS. A bias in gender was observed in the studies investigated the effects of tDCS on foot sensory function and foot muscle strength. However, to date, no studies have examined how gender influences the tDCS-induced effects on foot physical performance, which is worthwhile to be explored in the future. Besides, much more numbers of studies generally demonstrated that tDCS can induce benefits to functional performance. However, potential publication bias should be noted, that is, studies with positive results are more preferred to be reported. Negative results on the efficacy of tDCS might be encouraged to be published to critique the implementation of tDCS, which will ultimately help optimize the protocol of tDCS intervention.

Research efforts on exploring the effects of tDCS on the physical performances of the foot and ankle have emerged recently, and a relatively limited number of studies (*n* = 8) was published. The current review was thus performed based upon this number of publications. Besides, this study only focused on the effect of tDCS on the physical performances of the foot and ankle of healthy adults; patients with diminished or impaired foot and ankle functionality should be taken into account in future studies.

## Conclusion

Based on the existing studies, tDCS is promising to help improve the physical performances of the foot and ankle, such as motor performance, motor learning skill, and somatosensory function, which needs to be examined and confirmed in future studies. The underlying mechanism has been postulated that tDCS increased neuronal excitability of the targeted cortex and may potentially enhance the neuromechanical management of the physical performances of the foot and ankle. However, future studies with a larger sample size and a more rigorous experimental design (i.e., study protocol and tDCS montage) are warranted to confirm the findings of current studies and explore the neurophysiological and neuromechanical mechanisms of tDCS-induced improvements in the physical performances of the foot and ankle.

## Data Availability Statement

The original contributions presented in the study are included in the article/supplementary materials, further inquiries can be directed to the corresponding authors.

## Author Contributions

SX contributed to study design, data collection, drafting, and revising the manuscript. JZ and WF contributed to supervising study design, completing data analysis and interpretation, and revising the manuscript. BW and XZ revised the manuscript. All authors have read and approved the final version of the manuscript and agree with the order of the presentation of the authors.

## Conflict of Interest

The authors declare that the research was conducted in the absence of any commercial or financial relationships that could be construed as a potential conflict of interest.
